# Misperception of weight status in the pacific: preliminary findings in rural and urban 11- to 16-year-olds of New Caledonia

**DOI:** 10.1186/s12889-016-3982-0

**Published:** 2017-01-05

**Authors:** Stéphane Frayon, Sophie Cherrier, Yolande Cavaloc, Guillaume Wattelez, Amandine Touitou, Paul Zongo, Kalina Yacef, Corinne Caillaud, Yannick Lerrant, Olivier Galy

**Affiliations:** 1 Interdisciplinary Laboratory for Research in Education, School of Education, University of New Caledonia, Noumea, New Caledonia; 2 Laboratory ACTES, EA 3596, UFR STAPS Antilles, Pointe à Pitre, French West Indies Guadeloupe; 3 School of Information Technologies, University of Sydney, Sydney, Australia; 4 Faculty of Health Sciences and Charles Perkins Centre, University of Sydney, Sydney, Australia

**Keywords:** Weight perception, Overweight, Adolescents, Ethnicity, Polynesian, Melanesian

## Abstract

**Background:**

Adolescent obesity is prevalent in Pacific region ethnic groups (European, Melanesian and Polynesian) living in both urban and rural areas. Although body perception is an important factor of weight gain or loss, little is known about the body self-perceptions of Pacific region adolescents. This study therefore evaluated adolescent perceptions of body weight according to ethnicity (European, Melanesian or Polynesian), socioeconomic status (low, intermediate or high) and living area (rural or urban) in New Caledonia.

**Methods:**

Sociodemographic and anthropomorphic data from 737 adolescents (351 boys and 386 girls) with ages ranging from 11 to 16 years were collected and analysed. The International Obesity Task Force (IOTF) standards were used to define weight status as normal-weight, underweight or overweight/obese. Weight perception was assessed from detailed questionnaires, with adolescents rating their own weight with the following descriptors: ‘about the right weight’, ‘too heavy’, or ‘too light’.

**Results:**

Results showed that only 8.5% of normal-weight adolescents (7% boys and 10% girls) identifying themselves as ‘too heavy’. Normal-weight Melanesian adolescents were less likely than their European counterparts to assess themselves as too heavy (OR = 0.357). However, half the overweight/obese adolescents underestimated their weight status (53% boys and 48% girls). Weight misperception was associated with ethnicity, socioeconomic status and living area, with gender-specific differences.

**Conclusions:**

The results of this study suggest that these sociodemographic factors should be taken into account when designing public health policies and health education school programmes in New Caledonia and, more broadly, the Pacific region.

## Background

Excessive weight is mainly associated with non-communicable diseases like cardiovascular events, type 2 diabetes mellitus and hypertension [1]. When present from very early childhood (juvenile obesity), obesity may cause physiological and psychological disorders in adulthood [2]. A recent study showed an upward trend in global obesity since 1975 and estimated that the prevalence would reach 18% in men and 21% in women by 2025, with severe obesity surpassing 6% in men and 9% in women [3]. In addition, the percentage of overweight adolescents that remain so into adulthood ranges from 22 to 90%, depending on the study [4].

In the Pacific region, globalisation, trade liberalisation and increasing urbanisation have all contributed to a shift in nutrition and physical activity [5]. Traditional diets of root crops, vegetables, fruits, and fresh fish and meat have been steadily replaced by imported and processed energy-dense, low-nutrient foods. Since 2005, these environmental changes have had an increasingly strong impact on younger generations [6], with one result being the high prevalence of overweight and obesity in adolescents today [7]. Adolescents therefore seem particularly exposed to the risk of becoming overweight [8, 9] and its psychological consequences [8, 10]. Yet, high body mass index (BMI) and high body fat mass (BFM) (mean value: 24.24 kg.m^−2^ ± 2.7 and 17.6% ± 2.4, respectively) were recently observed in young Melanesian athletes living in New Caledonia, suggesting that the Pacific lifestyle and/or genetic factors, even with high levels of physical activity and sport performance, have an impact on weight status [11]. For the most part, the New Caledonian population has an economic status in line with that of Western countries, but half the population nevertheless follows a traditional tribal lifestyle, similar to that of other Melanesians in the Pacific (i.e. Papua New Guinea [12]). In fact, most Melanesian people in rural areas still live in tribal communities that drive physical activity (fishing/cropping/hunting) and food-consumption behaviours (consuming tubers like manioc or ignam, fruits and fish), even when some Westernisation is evident.

Many factors play a role in weight gain or loss, and the self-perception of body mass is an important factor. Self-perception of body mass is defined as the subjective appraisal of one’s own weight status, and an accurate is crucial for the success of overweight prevention programmes. For example, normal-weight teens who perceive themselves as overweight may make unnecessary efforts to lose weight, which, in extreme cases, can lead to eating disorders [13, 14]. On the other hand, overweight adolescents who fail to recognise their excess weight may underestimate the need for a healthier diet and physical activity [15, 16]. Misperceptions are common in populations around the world, although notably in the United States, where 38% of overweight and 8% of obese people perceive themselves as normal weight [17]. Several factors contribute to this misperception, including education level, ethnicity and socioeconomic status [18]. For example, lower socioeconomic and education levels are associated with greater discrepancies between real and perceived body weight [17, 19, 20]. In a New Zealand study, overweight or obese people of Pacific origin were more likely than people of European origin [19] to think that their body size was normal. These findings suggest that weight management interventions should take ethnicity into consideration, and this may be particularly important in New Caledonia, which has a culturally and ethnically diverse population, including people from many of the ethnic communities present in the Pacific (predominantly Melanesian, Polynesian, Asian and European).

Studies in adolescent populations that have examined the frequency of overestimation and underestimation of body size have reported that up to 25% of normal-weight girls and about 5% of normal-weight boys perceive themselves as overweight [20]. Moreover, up to 48% of overweight girls and 61% of overweight boys perceive themselves as normal-weight [16, 20, 21]. However, most of these studies were based on data collected in the United States or Europe and cannot be generalised to other populations, especially to Pacific islanders [11].

Other studies have shown a difference in the prevalence of overweight/obesity in rural compared with urban areas [22], although whether the place of residence (rural versus urban) influences perception of body size is still under debate. While some studies found no difference in body size perception according to place of residence [23], others showed a correlation between place of residence (rural or urban) and misperception of weight status. For example, a recent work showed that Korean girls living in a rural area were less likely to overestimate their weight than those living in an urban area [24].

Given the high prevalence of overweight in Pacific adolescents [7], larger body sizes might be considered as normal in this region, with overweight status having a higher threshold than in other parts of the world: children and adolescents living in environments in which their relatives, parents and schoolmates are overweight or obese may develop misperceptions about what constitutes appropriate weight status [25]. On the other hand, because overweight is a major public health problem in the Pacific region, health information on this topic is frequently and widely communicated by the media and public health agencies. These messages aim to improve adolescents’ overall health literacy, especially regarding obesity, and raise awareness about the dangers of excess weight among those who are overweight/obese. To our knowledge, self-perception of body weight in New Caledonian adolescents is still poorly understood.

This study explored whether place of residence influences body weight perception in adolescents living in New Caledonia. In particular, we investigated the proportion of normal-weight adolescents (11–16 years old) who consider themselves overweight (overestimation) or underweight (underestimation), and those overweight/obese adolescent who consider themselves normal-weight (underestimation), according to their living area.

## Methods

### Data and participants

Our study was part of a community-based obesity prevention project conducted in selected representative school sites in the three Provinces of New Caledonia. The aim is to prevent obesity by encouraging healthy eating and physical activity. The study was conducted at the schools during classe time.

Sample size was calculated using OpenEpi software for epidemiologic statistics version 3.01, with a confidence (1-α) of 95% and margin of error of ±0.05. Because misperception of body weight varied among weight status categories, anticipated prevalence was assumed to be 50%, as this magnitude yielded the maximum sample size. The minimum sample size calculated was 384. The sample size was then increased by 10–15% to account for missing data.

New Caledonia is divided into three provinces (North Province, South Province and Loyalty Islands Province), all substantially different in terms of ethnic distribution, SES and urbanisation. Five secondary public schools were selected: one in Islands Province (rural area), two in North Province (east and west coasts, rural areas) and two in South Province in the capital, Noumea, which is the only urban zone of New Caledonia. This selection yielded a representative repartition between rural and urban areas, which is 63 and 37% respectively in New Caledonian. The selection criterion was school size (*n* > 200) to ensure sufficient data in a single field trip. Based on this criterion, only one school was eligible in Islands Province, and four schools were eligible in North Province (two on each coast) and eight in South Province. Schools were then randomly selected and contacted to obtain staff agreement (principal and teacher), including that of the school nurse. Two classes were then randomly selected in each of four grades (levels) by the school’s Principal, for a total of 180 students (8 groups with a mean of 22.5 students per division). As the schools in the urban area were rather large (*n* > 500), collecting data was more difficult (availability of nurses or rooms, incompatible timetables) and data from only six divisions were finally obtained. In each school, only 90% of the data was obtained due to student absences or parental refusal. Adolescents with missing data or those from too small ethnic groups were then excluded. The proportion of adolescents with missing data or from minor ethnic groups was higher in the urban area (5.5%) than the rural areas (2.5%). Finally, this study included 737 adolescents (11–16 years old).

### Anthropometric parameters

All anthropometric data were collected by trained staff in the school nurse’s office. Height was measured to the nearest 0.1 cm using a portable stadiometer (Leicester Tanita HR 001, Tanita Corporation, Tokyo, Japan). Weight was determined to the nearest 0.1 kg using a scale (Tanita HA 503, Tanita Corporation, Tokyo, Japan), with adolescents weighed in light clothing. Body mass index (BMI) was calculated by dividing weight in kilograms by height squared in meters.

The BMI standard deviation score (BMI-SDS) and percentile were calculated by the LMS method using the International Obesity Task Force (IOTF) reference values from Cole et al. [26]. Weight status was defined according to the IOTF criteria [26], which are used to classify BMI values according to age and gender as thin (underweight), normal weight, overweight, or obese, based on adult BMI cutoffs at 18 years. Because the normal-weight category covers a large spectrum of weight from ‘almost thin’ to ‘almost overweight’, for some analyses we divided the ‘normal weight’ category into two categories: BMI-SDS below 0.0 (termed ‘lower normal weight’) and BMI-SDS above 0.0 (termed ‘upper normal weight’), as previously described [27].

### Sociodemographic characteristics

The demographic information used in the analyses included age, gender, ethnicity, and socioeconomic status (SES). Ethnicity was self-reported by the adolescents and categorised as recommended in the INSERM report on New Caledonia [28] using an anonymous survey tool. SES was indexed on the basis of the occupation of the household reference person (defined as the householder with the highest income) using the National Statistics Socio-Economic Classification [29]. For the present analyses, we generated three categories: managerial and professional occupations (higher SES), intermediate occupations (intermediate SES), and routine and manual occupations (lower SES).

For the data from the last census in New Caledonia [30], the degree of urbanisation was determined using a European standard [31]. Densely populated areas comprising at least 50,000 inhabitants in a continuous zone with more than 500 inhabitants per km^2^ were classified as urban. Semi-urban areas were defined as continuous areas with more than 50,000 inhabitants, over 100 inhabitants per km^2^, and adjacent to an urban area. Rural areas were those that did not fulfil the conditions for an urban or semi-urban area.

### Weight perception

The adolescents completed an online survey during school hours about their food and nutrition behaviours, physical activity behaviours and weight self-perceptions. Weight perception was assessed with the question: ‘Given your age and height, would you say that you are about the right weight, too heavy, or too light?’ [27].

### Statistics

All analyses were conducted using SPSS Version 22, with a *P* value < .05 indicating statistical significance. We conducted analyses separately for boys and girls. The gender differences were assessed using *t*-tests (continuous variables) or chi^2^ analyses (categorical variables). We used multivariate logistic regressions to identify the factors associated with overestimation or underestimation of weight status. The variables in the models were age, gender, ethnicity, SES residence (urban vs. rural) and weight status (lower vs. upper normal-weight in normal-weight respondents; overweight vs. obese in overweight/obese respondents).

## Results

Table 1 shows the overall descriptive data for the whole sample and for boys and girls. The sample included 351 boys and 386 girls from 11 to 16 years old (13.7 ± 1.5 years). The repartition by socioeconomic status indicated that 44.2% participants were in the low SES category, while 30.9% were in the high category and 24.8% in the intermediate category. The majority of adolescents had a BMI that placed them in the normal-weight category; however, 21.2% were overweight and 12.5% were obese. Boys were taller than girls on average, but there were no significant gender differences regarding weight, weight status or other sociodemographic characteristics.

**Table 1:** Demographic and anthropometric characteristics of the 11- to 16-year-olds in this study, overall and by gender – mean (SD) or % (n)

	Whole sample	Boys	Girls	P*
	(*n* = 737)	(*n* = 351)	(*n* = 386)	
Age (years)	13.66 (1.48)	13.63 (1.55)	13.69 (1.42)	.644
Ethnicity % (n)				
European	34.5 (254)	34.2 (120)	34.7 (134)	.893
Melanesian	60.2 (444)	60.1 (211)	60.4 (233)	-
Polynesian	5.3 (39)	5.7 (20)	4.9 (19)	-
SES % (n)				
Higher	30.9 (228)	32.2 (113)	29.8 (115)	.056
Intermediate	24.8 (183)	27.9 (98)	22.0 (85)	-
Lower	44.2 (326)	39.9 (140)	48.2 (186)	-
Residence % (n)				
Urban	27.7 (204)	30.8 (108)	24.9 (96)	.074
Rural	72.3 (533)	69.2 (243)	75.1 (290)	-
Height (cm)	158.42 (9.50)	159.67 (11.25)	157.28 (7.41)	.001
Weight (kg)	55.46 (14.60)	55.50 (15.37)	55.42 (13.89)	.938
BMI-SDS	0.792 (1.15)	0.78 (1.18)	0.80 (1.13)	.816
BMI centile	69.35 (27.97)	68.47 (28.03)	70.15 (27.94)	.415
Weight status % (n)				
Underweight	5.2 (38)	4.3 (15)	6.0 (23)	.176
Lower normal weight^a^	20.4 (150)	23.1 (81)	17.9 (69)	-
Upper normal weight^a^	40.8 (301)	41.6 (146)	40.2 (155)	-
Overweight	21.2 (156)	18.2 (64)	23.8 (92)	-
Obese	12.5 (92)	12.8 (45)	12.2 (47)	-

Mean +/− SD or % (n)

*SES* socioeconomic status, *BMI-SDS* body mass index standard deviation score

*P values are for the association between each variable and gender

^a^‘Lower normal-weight’ and ‘upper normal-weight’ categories are subcategories of the normal-weight category

### Weight status perception

**Fig. 1:**
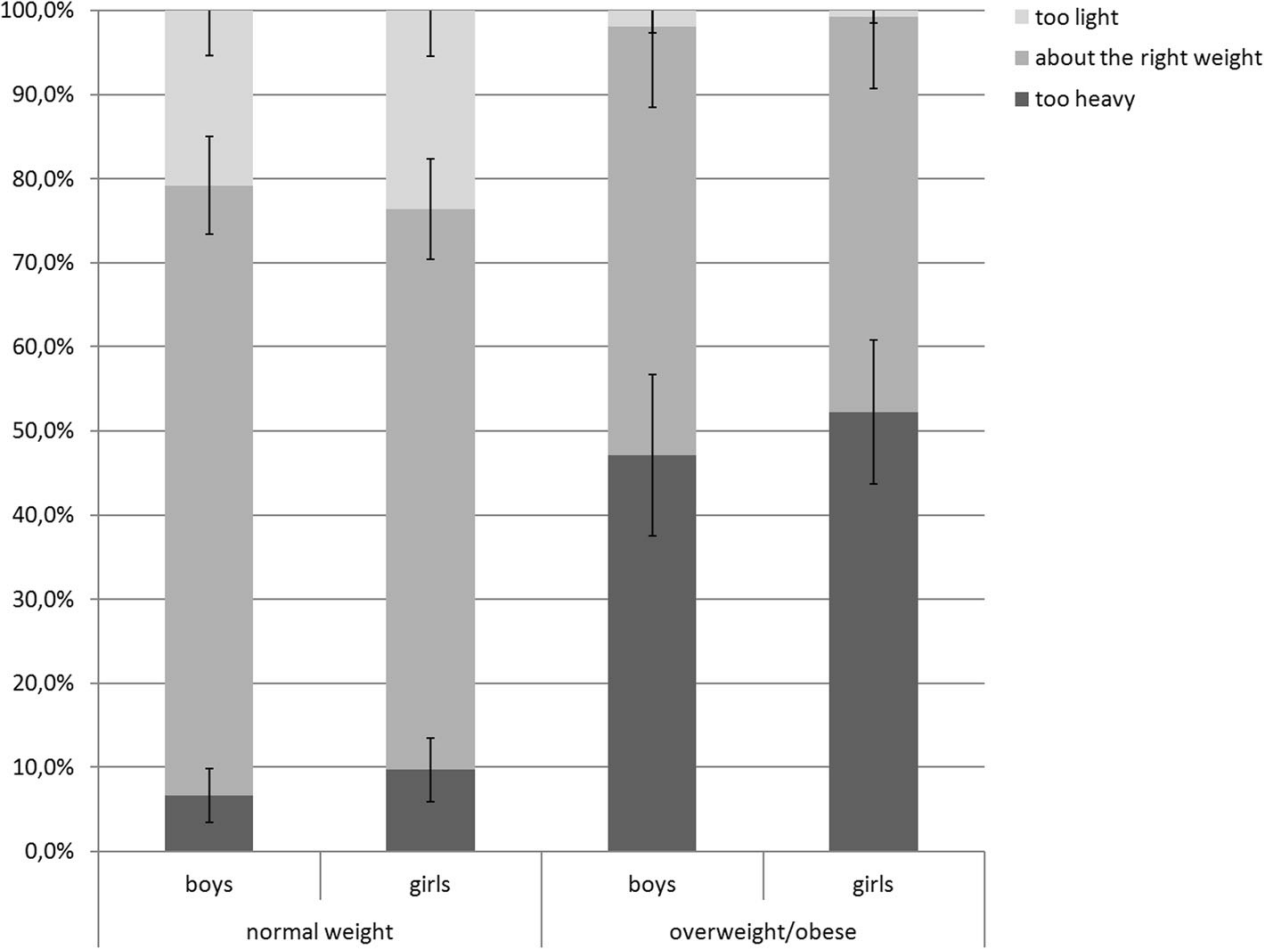
The proportion (with 95% CI) of boys and girls who reported feeling ‘too heavy’, ‘about the right weight’, and ‘too light’, by measured weight status

Figure 1 presents the perception of weight status by gender and actual weight status. Weight status perception errors were frequent, as they concerned 35.2% of the adolescents in our sample (Fig. 1). There was no difference between the rate of misperception between boys and girls (34.2 vs. 36.0%, respectively, *P* = .619). Of the normal-weight adolescents, 69.5% correctly identified themselves as about the right weight, but 8.2% thought they were too heavy and 22.2% thought they were too light. Of the overweight/obese adolescents, 50.0% felt they were too heavy, but 48.7% thought they were about the right weight and 1.3% felt they were too light. Again, there was no difference between girls and boys for this misperception (*P* = .571).

### Body perception among normal-weight adolescents: overestimation

The factors independently associated with an overestimation of weight status (feeling ‘too heavy’) among normal-weight participants were extracted with a multivariate analysis. Results are shown in Table 2. Melanesian adolescents of normal weight were less likely to self-assess as too heavy compared with their European counterparts (OR = 0.357, *P* = .033). Low and intermediate SES normal-weight boys were more likely than high SES boys to think that they were too heavy, although this difference was only significant in the intermediate category (low SES boys OR = 5.396, *P* = .084; intermediate SES boys: OR = 6.431, *P* = .034). This difference is not found among girls (low SES girls: OR = 1.041, *P* = .952; intermediate SES girls: OR = 2.031, *P* = .246).

**Table 2:** Predictors of size overestimation (feeling ‘too heavy’) among normal-weight adolescents (multivariate analysis^c^)

	Whole sample (*n* = 429)			Boys (*n* = 212)			Girls (*n* = 217)		
	%^a^ (n)	OR [95% CI]	*P*	%^a^ (n)	OR [95% CI]	*P*	%^a^ (n)	OR [95% CI]	*P*
Age (years)^b^	-	1.45 [1.07–1.96]	.017	-	2.195 [1.283–3.753]	.004	-	1.035 [0.691–1.551]	.869
Gender									
Female	10.1 (22)	1.00		-	-	-	-	-	-
Male	7.1 (15)	0.648 [0.311–1.352]	.248	-	-	-	-	-	-
Ethnicity									
European	13.3 (22)	1.00		11.5 (9)	1.00		14.9 (13)	1.00	
Melanesian	4.3 (7)	0.357 [0.147–0.868]	.023	3.1 (4)	0.222 [0.043–1.159]	.074	5.6 (7)	0.468 [0.158–1.383]	.170
Polynesian	36.4 (4)	3.696 [0.883–15.473]	.074	28.6 (2)	2.568 [0.319–20.649]	.375	50.0 (2)	5.915 [0.675–51.823]	.108
SES									
Higher	6.2 (9)	1.00		6.6 (5)	1.00		13.0 (9)	1.00	
Intermediate	11.2 (12)	2.793 [1.085–7.186]	.033	8.3 (5)	6.431 [1.153–35.864]	.034	14.9 (7)	2.031 [0.613–6.736]	.246
Lower	9.7 (17)	1.942 [0.700–5.389]	.202	6.6 (5)	5.396 [0.799–36.424]	.084	5.9 (6)	1.041 [0.280–3.870]	.952
Residence									
Urban	17.6 (22)	1.00		15.4 (10)	1.00		20.0 (12)	1.00	
Rural	4.9 (15)	0.509 [0.192–1.350]	.175	3.4 (5)	0.479 [0.087–2.626]	.397	6.4 (10)	0.433 [0.125–1.500]	.187
Weight status^d^									
Upper-normal weight	9.2 (26)	1.00		9.0 (12)	1.00		9.3 (14)	1.00	
Lower-normal weight	7.6 (11)	0.519 [0.230–1.170]	.114	3.8 (3)	0.197 [0.039–0.990]	.049	11.9 (8)	0.930 [0.344–2.515]	.886

*OR* odds ratio, *CI* confidence interval, *SES* socioeconomic status

^a^Indicates the percentage of normal-weight participants in each group perceiving themselves to be too heavy

^b^Entered into the model as a continuous variable

^c^Variables in the models are: age (years), gender, ethnicity, SES, residence and weight status category

^d^‘Lower normal weight’ and ‘upper normal weight’ category were subcategories of the normal weight category

The odds of overestimation increased significantly with age in boys only (OR = 2.195, *P* = .004). Last, the boys in the lower-normal BMI category were less likely to feel ‘too heavy’ than their upper-normal BMI counterparts (OR = 0.197, *P* = .049). This difference was not found among girls (OR = 0.930, *P* = .886). Place of residence and gender were independently associated with overestimation of body weight.

### Body perception among normal-weight adolescents: underestimation

Table 3 shows the multivariable models to examine the factors independently associated with underestimation of body weight in the normal-weight boys and girls (self-identification as ‘too thin’). Underestimation was more likely in the lower normal-weight group (29.0%) than in the upper normal-weight group (13.4%), with significant effects in both genders (boys: odds ratio [OR] = 4.043, *P* = <.001; girls: OR = 3.306, *P* = .003). The place of residence increased the risk of misperception but differed greatly by gender. Normal-weight girls in rural areas were significantly more likely than normal-weight girls in urban areas to consider themselves to be ‘too thin’ (24.8% vs. 5.0%, OR = 5.264, *P* = .021). Among normal-weight boys, those who lived in rural areas tended to perceive themselves as ‘too thin’ less often than those living in cities, but the difference was not significant (OR = 0.603; *P* = .317). Low and intermediate SES normal-weight adolescents were more likely than high SES adolescents to think that they were too heavy (Low SES: OR = 2.137, *P* = .033; intermediate SES: OR = 2.122, *P* = .044). Ethnicity and age were not independently associated with underestimation of weight status.

**Table 3:** Predictors of size underestimation (feeling ‘too light’) among normal-weight adolescents (multivariate analysis^c^)

	Whole sample (*n* = 429)			Boys (*n* = 212)			Girls (*n* =217)		
	%^a^ (n)	OR [95% CI]	*p*	%^a^ (n)	OR [95% CI]	*p*	%^a^ (n)	OR [95% CI]	*p*
Age (years)^b^	-	0.892 [0.730–1.089]	.261	-	0.813 [0.621–1.065]	.133	-	1.068 [0.778–1.468]	.683
Gender									
Female	19.4 (42)	1.00		-	-	-	-	-	-
Male	17.9 (17)	0.810 [0.481–1.363]	.427	-	-	-	-	-	-
Ethnicity									
European	12.7 (21)	1.00		15.4 (12)	1.00		10.3 (9)	1.00	
Melanesian	22.5 (57)	1.682 [0.906–3.124]	.100	18.9 (24)	1.390 [0.568–3.398]	.470	26.2 (33)	1.989 [0.823–4.805]	.127
Polynesian	18.2 (2)	1.590 [0.287–8.814]	.596	28.6 (2)	1.748 [0.258–11.836]	.567	-	nd	-
SES									
Higher	11.0 (16)	1.00		13.2 (10)	1.00		8.7 (6)	1.00	
Intermediate	21.5 (23)	2.122 [1.020–4.416]	.044	25.0 (15)	2.342 [0.888–6.174]	.085	17.0 (8)	2.017 [0.610–6.674)	.250
Lower	23.2 (41)	2.137 [1.063–4.296]	.033	17.1 (13)	1.674 [0.588–4.760]	.334	27.7 (28)	2.723 [0.976–7.596]	.056
Residence									
Urban	12.0 (1)	1.00		18.5 (12)	1.00		5.0 (3)	1.00	
Rural	21.4 (65)	1.360 [0.638–2.901]	.426	17.7 (26)	0.603 [0.224–1.623]	.317	24.8 (39)	5.264 [1.287–21.532]	.021
Weight status^d^									
Upper-normal weight	13.4 (38)	1.00		10.4 (14)	1.00		16.0 (24)	1.00	
Lower-normal weight	29.0 (42)	3.442 [2.025–5.851]	<.001	30.8 (24)	4.043 [1.893–8.634]	<.001	26.9 (18)	3.306 [1.489–7.338]	.003

*OR* odds ratio, *CI* confidence interval, *SES* socioeconomic status

^a^Indicates the percentage of overweight participants in each group perceiving themselves to be too light

^b^Entered into the model as a continuous variable

^c^Variables in the models are: age (years), gender, ethnicity, SES, residence and weight status category

nd: not determined due to small size of the subgroup

^d^‘Lower normal weight’ and ‘upper normal weight’ category were subcategories of the normal weight category

### Body perception among overweight/obese adolescents: underestimation

Table 4 shows the multivariate models to examine the factors independently associated with underestimation of body weight in the overweight/obese boys and girls (identifying themselves as ‘too thin’ or ‘normal weight’). Among overweight non-obese girls, it was more common for them to believe they were of normal weight than being overweight (OR = 3.774, *P* = .002). This trend was not significant for boys (OR = 2.169, *P* = .075). SES played a role for boys because low SES increased the odds of self-perceiving normal weight (OR = 4.113, *P* = .018), which was not the case for the girls (OR = 1.353, *P* = .538). The perception of being normal-weight was independent of gender, place of residence or age.

**Table 4:** Predictors of size underestimation (feeling ‘about the right weight’ or ‘too light’) among overweight/obese adolescents (multivariate analysis^c^)

	Whole sample (*n* =236)			Boys (*n* = 104)			Girls (*n* =132)		
	%^a^ (n)	OR [95% CI]	*P*	%^a^ (n)	OR [95% CI]	*P*	%^a^ (n)	OR [95% CI]	*P*
Age (years)^b^	-	0.930 [0.760–1.137]	.480	-	1.096 [0.815–1.472]	.545	-	0.837 [0.625–1.120]	.232
Gender									
Female	47.7 (63)	1.00		-	-	-	-	-	-
Male	52.9 (55)	1.366 [0.790–2.362]	.265	-	-	-	-	-	-
Ethnicity									
European	47.2 (25)	1.00		60.9 (14)	1.00		36.7 (11)	1.00	
Melanesian	52.8 (53)	1.082 [0.555–2.107]	.817	50.7 (36)	0.640 [0.228–1.791]	.395	54.4 (49)	1.406 [0.540–3.659]	.485
Polynesian	36.4 (8)	0.844 [0.272–2.617]	.769	50.0 (5)	0.847 [0.157–4.568]	.847	25.0 (3)	0.748 [0.142–3.954]	.733
SES									
Higher	38.0 (19)	1.00		35.0 (7)	1.00		40.0 (12)	1.00	
Intermediate	47.6 (30)	1.562 [0.695–3.511]	.281	48.5 (16)	2.144 [0.645–7.124]	.213	46.7 (14)	1.296 [0.396–4.237]	.668
Lower	56.1 (69)	2.068 [1.005–4.254]	.048	62.7 (32)	4.113 [1.276–13.255]	.018	51.4 (37)	1.353 [0.517–3.543]	.538
Area of residence									
Urban	39.5 (15)	1.00		52.4 (11)	1.00		23.5 (4)	1.00	
Rural	52.0 (103)	1.491 [0.658–3.381]	.339	53.0 (44)	0.826 [0.275–2.478]	.733	51.3 (59)	2.773[0.711–10.812]	.142
Weight status									
Obese	34.9 (52)	1.00		41.5 (26)	1.00		28.9 (25)	1.00	
Overweight	58.7 (50)	2.975 [1.667–5.311]	<.001	60.3 (25)	2.169 [0.926–5.082]	.075	57.5 (26)	3.774 [1.649–8.636]	.002

*OR* odds ratio, *CI* confidence interval, *SES* socioeconomic status

^a^Indicates the percentage (and effective) of overweight participants in each group perceiving themselves to be about the right weight or too light

^b^Entered into the model as a continuous variable

^c^Variables in the models are: age (years), gender, ethnicity, SES, residence and weight status category

## Discussion

### Main findings

This study investigated self-perception of weight status in New Caledonian adolescents of the Pacific region. Misperception of weight status was found in 30.5% of the normal-weight adolescents and 50% of the overweight/obese adolescents, who underestimated their weight. This was particularly the case for overweight boys from low SES. We also observed that normal-weight girls from rural areas were more likely to underestimate their weight status and to consider themselves as ‘too thin’.

These findings show that in New Caledonia, weight underestimation is frequent in overweight and obese adolescents and less frequent in those of normal weight. These findings are consistent with the results of several previous studies of adolescent weight perceptions in Europe and the United States [20, 21, 27].

### Comparison with other studies

Normal-weight Melanesian adolescents seemed less likely than their European counterparts to consider themselves ‘too heavy’ (4.3% vs. 13.3%). These results are consistent with those obtained in the United States, where ethnicity influences adolescent self-perceptions of weight status [32]. In this latter study, Martin showed that African-Americans were less likely to overestimate their weight than their European-American counterparts. For overweight and normal-weight adolescents, however, there was no significant difference in underestimation (feeling ‘too light’) of weight status according to ethnicity. A previous study also showed that overestimation of weight status in normal-weight adolescents can lead to unhealthy weight-control practices [13]. Our findings suggest a lower risk of these behaviours in Melanesians compared with European adolescents, but this hypothesis needs further investigation.

It is worth noting that there was no significant difference in our sample between Polynesian and European adolescents concerning overestimation or underestimation. It has been suggested that the high rates of overweight in the Polynesian population could be related to a poor perception of their own weight status, especially during adolescence [11]. Our results indicate that misperceptions of weight status are not more common in adolescents from Polynesian origin than they are in other populations.

The adolescents from the low SES category were more likely to consider themselves ‘too thin’ when they were in the normal-weight, overweight and obese categories. The relationship between socioeconomic status and self-perception of weight status has been demonstrated in several studies [17, 33–36]. The relationship has been explained by differences in the definition of ‘normal’ or ‘ideal’ weight among SES groups [37] and the observation that individuals with high SES tend to be more weight-conscious and to have greater access to health information [38].

The normal-weight girls in rural areas were more likely than those in urban areas to consider themselves too thin (underestimation) (24.8% vs. 5.0%). These findings probably indicate greater societal pressure towards thinness [39, 40] for the girls living in urban areas.

Gender alone had no impact on the misperception of weight status in our study. However, in the subgroups, some effects associated with misperception were gender-dependent. For example, age increased the overestimation of normal weight twofold for boys but not for girls. Normal-weight girls in rural areas were more likely to consider themselves too thin than those living in urban areas, but no significant difference was observed between boys from rural and urban areas. It is well known that some misperceptions are more prevalent in girls compared with boys. In general, girls are more likely than boys to overestimate and less likely to underestimate their body weight [24]. Accurate self-perception of weight status needs a well-calibrataed reference image. For adolescent girls, thinness has become the ideal body type, and this often unrealistic body type is the standard by which many of these adolescents define themselves and others [41]. Given that female bodily attractiveness is associated with an unrealistic thin body type, many adolescent girls feel overweight with a normal weight [42].

During adolescence, the ideal-body image is determined by a variety of environmental, social, physical, and psychological factors. The media have one of the strongest influences on body image today, and they often target those teenagers and peers who help shape beliefs about the ideal body [41]. In New Caledonia, it is likely that the adolescents living in rural areas and Melanesians are less influenced by these stereotypical images of ideal body. The lack of cinemas in the rural areas of New Caledonia, the relative dearth of magazines and generally a more limited access to various media may explain these differences. Recently, Tiggemann and Slater [43] found that weight control increases with time spent on the Internet and social networks (for instance, Facebook) among 13- to 15-year-old girls. In this respect, the situation of young people in New Caledonia varies widely since youth between 15 and 29 years living in urban areas are more likely (88%) to connect to social networks than those in North Province (70%) or Loyalty Islands Province (55%) [44]. We can also hypothesise that the environment in rural areas may have altered the adolescents’ self-perceptions of overweight: a high overweight rate among schoolmates and relatives tends to normalise overweight [25]. Therefore, the perception of being in a normal-weight category may have reflected what they considered an acceptable standard at the time they completed the questionnaire.

One of the strengths of our study was the use of actual height and weight to calculate BMI-based weight status, as several studies have observed that BMI based on self-reported height and weight is underestimated [28, 29]. However, some limitations should be noted. First, we used BMI reference values from Cole et al. to determine weight status in our sample. Yet a recent review and meta-analysis showed a pooled sensitivity of 0.73 and a pooled specificity of 0.93 when a BMI-based reference value was used against body fat mass to detect overweight adolescents [45]. Misclassification of the adolescents may thus have modified the misperception prevalence in this study. To lend support to our results, the self-perception of weight status should be compared against another reference like body fat contents. Another limitation of this study is the small sample size, although it represents 3.2% of the adolescents within the same age range enrolled in school. In particular, only 39 of the adolescents were of Polynesian origin (5.3%), whereas Polynesian adolescents make up 12.2% of the 10- to 19-year-olds in whole population [30].. Similarly, urban adolescents made up 27.7% of the adolescents in this study, whereas they make up 37% of the New Caledonians living in the urban area of Noumea. Last, we investigated adolescents from 11 to 16 years old and we therefore do not know how misperceptions of body weight evolve in older adolescents. Despite these limitations, our results are consistent with several international studies, which suggest that these methodological concerns did not bias the results.

### Consequences for public health programmes

The differences in weight status perceptions in New Caledonia between rural and urban populations and among ethnicities are crucial factors for tailoring effective obesity prevention programmes. Self-awareness of being overweight is an important factor in attempting to lose weight [46], and we therefore think that individual weight status should be determined for each adolescent in the schools, with the information given to both the parents and their children. The messages to prevent obesity in adolescents also need to be carefully thought out. For example, among adolescents who think they are overweight when they are not, which was more frequent in European than Melanesian adolescents, the risk is that inappropriate behaviour will be encouraged [47]. Indeed, previous studies have indicated that problems with weight perception among normal-weight teens can lead to unnecessary and sometimes unhealthy types of dieting behaviours [14] and in extreme cases can cause eating disorders [48]. Recently, Van Vliet [49] showed that feeling too fat rather than being too fat increases unhealthy eating habits among adolescents, usually in the direction of reduced food intake. These observations suggest that the focus of public health initiatives should not be limited to weight loss and obesity prevention, but should also take into consideration body image, as noted by Voelker in his recent review [41]. Programmes that promote a healthy body image for both adolescent boys and girls are needed in New Caledonia, but they will need to take into account the specificities of this multi-ethnic population. Multisession interventions focused on media literacy, boosting self-esteem, and building peer support seem to have the most effective results [50].

Individuals with high SES are more weight-conscious [38] and more likely to recognise excess weight, and therefore interventions that aim to address individual weight perceptions should initially target lower SES groups.

## Conclusions

This study showed that 50% of the overweight/obese adolescents living in New Caledonia underestimated their weight status. Weight misperception was associated with sociodemographic factors such as place of residence, SES and ethnicity, with some gender-specific differences. Overweight boys from low SES were particularly prone to underestimating their weight status. Normal-weight girls from rural areas were also more likely to underestimate their weight status and consider themselves to be ‘too thin’.

These differences in body weight perception and socio-environmental factors should be taken into account in the implementation of health promotion initiatives targeting children and teens in New Caledonia. In addition, the results suggest that educational components that address risky behaviours to control weight should be added to the prevention programmes for these age groups.

## Abbreviations

BFM: Body fat mass
BMI: Body mass index
IOTF: International obesity task force
SES: Socioeconomic status
